# Neuromodulation Therapies in Heart Failure: A State-of-the-Art Review

**DOI:** 10.1016/j.jscai.2023.101199

**Published:** 2023-12-04

**Authors:** Mohit Pahuja, Khawaja Hassan Akhtar, Satyam Krishan, Yusra Minahil Nasir, Philippe Généreux, Stavros Stavrakis, Tarun W. Dasari

**Affiliations:** a Department of Medicine, Section of Cardiovascular Medicine, University of Oklahoma Health Sciences Center, Oklahoma City, Oklahoma; b Department of Medicine, University of Oklahoma Health Sciences Center, Oklahoma City, Oklahoma; c Department of Medicine, Section of Cardiovascular Medicine, Morristown Medical Center, Morristown, New Jersey; d Heart Rhythm Institute, Department of Medicine, University of Oklahoma Health Sciences Center, Oklahoma City, Oklahoma

**Keywords:** autonomic dysregulation, device, heart failure, innovation, neuromodulation

## Abstract

Heart failure (HF) continues to impact the population globally with increasing prevalence. While the pathophysiology of HF is quite complex, the dysregulation of the autonomic nervous system, as evident in heightened sympathetic activity, serves as an attractive pathophysiological target for newer therapies and HF. The degree of neurohormonal activation has been found to correlate to the severity of symptoms, decline in functional capacity, and mortality. Neuromodulation of the autonomic nervous system aims to restore the balance between sympathetic nervous system and the parasympathetic nervous system. Given that autonomic dysregulation plays a major role in the development and progression of HF, restoring this balance may potentially have an impact on the core pathophysiological mechanisms and various HF syndromes. Autonomic modulation has been proposed as a potential therapeutic strategy aimed at reduction of systemic inflammation. Such therapies, complementary to drug and device-based therapies may lead to improved patient outcomes and reduce disease burden. Most professional societies currently do not provide a clear recommendation on the use of neuromodulation techniques in HF. These include direct and indirect vagal nerve stimulation, spinal cord stimulation, baroreflex activation therapy, carotid sinus stimulation, aortic arch stimulation, splanchnic nerve modulation, cardiopulmonary nerve stimulation, and renal sympathetic nerve denervation. In this review, we provide a comprehensive overview of neuromodulation in HF.

## Highlights

• Autonomic dysregulation plays a critical role in the pathophysiology of chronic heart failure .
• Neuromodulation strategies serve as an important target in management of heart failure.
• Device-based therapies continue to hold promise in patients with heart failure.
• We discuss the clinical and preclinical data on device therapies for neuromodulation.

## Central Illustration

**Figure:**
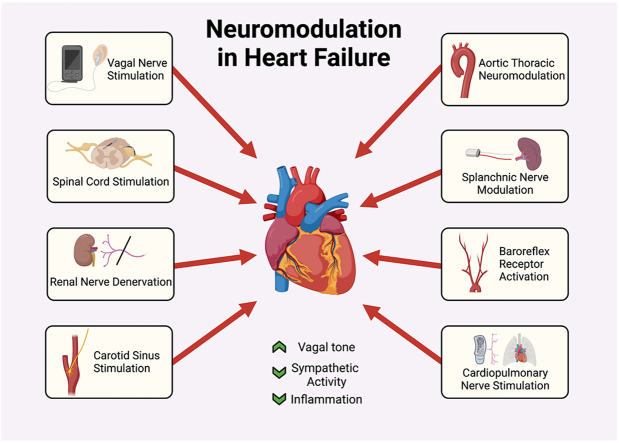

## Introduction

Despite availability of evidence-based therapies, heart failure (HF) continues to be the leading cause of morbidity and mortality in the United States and elsewhere.^1^, ^2^, ^3^ HF continues to impact the population globally and the prevalence of HF continues to rise; it is set to increase by 46% by 2030.^1^^,^^4^ Advanced HF therapies such as left ventricular assist devices and heart transplant have shown promising results but are not universally available and are reserved for a select few advanced HF patients. While the pathophysiology of HF is quite complex, the dysregulation of the autonomic nervous system, as evident in heightened sympathetic activity, serves as an attractive pathophysiological target for newer therapies and HF. In this review, we provide a comprehensive overview of device-based therapeutics targeting the autonomic nervous system in patients with HF syndromes.

## Pathophysiology of neurohormonal activation in HF

**Figure 1:**
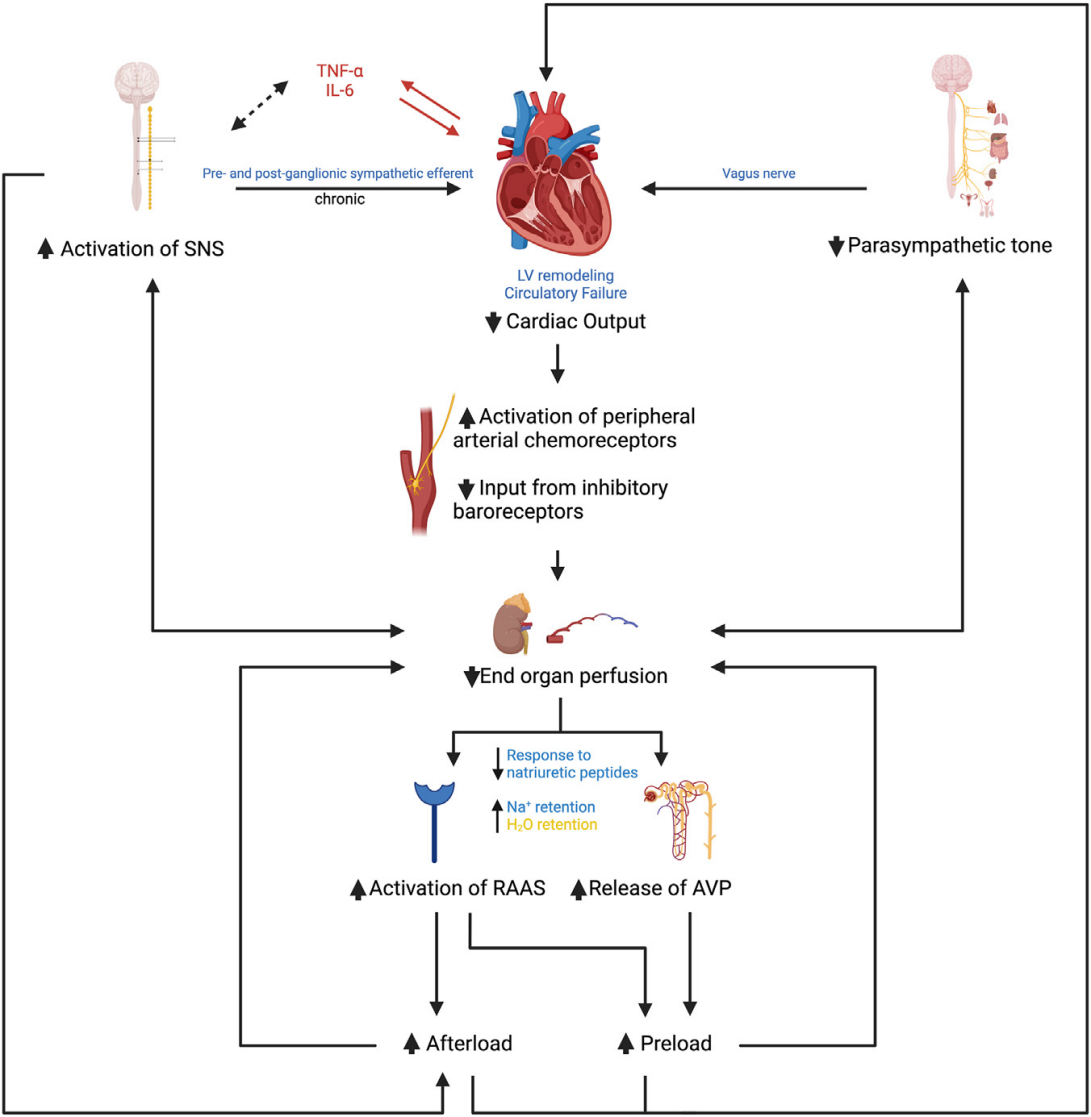
**Pathophysiology of neurohormonal activation in heart failure.** AVP, arginine vasopressin; IL-6, interleukin-6; LV, left ventricle; RAAS, renin-angiotensin-aldosterone system; TNF-α, tumor necrosis factor-α.

Earliest reports have implicated the upregulation of the sympathetic nervous system (SNS) in the pathophysiology and outcomes of HF.^5^ While the upregulation of the SNS can transiently support cardiovascular function during a compromised state, particularly in the acute HF syndrome, the sustained upregulation may have a deleterious impact on the clinical course of patients with HF. The degree of neurohormonal activation has been found to correlate to the severity of symptoms, decline in functional capacity, and mortality.^6^, ^7^, ^8^ The exact mechanism by which these neurotransmitters and inflammatory mediators contribute to the pathogenesis of HF is still poorly understood; however, it is widely believed that this neurohormonal activation occurs as a compensatory mechanism partly in response to a decline in cardiac function as an index event.^9^ These compensatory mechanisms include a complex interplay of neurohormonal and inflammatory markers that result in autonomic dysregulation involving a dysregulated balance between the SNS and parasympathetic nervous systems (PNS) (Figure 1).^10^ These compensatory mechanisms occur in response to low cardiac output, worsening end-organ perfusion, and low blood pressure, contributing to ventricular remodeling and dysfunction. Thus, autonomic dysregulation has a major role in the development of cardiac dysfunction and not entirely its sequelae.^9^^,^^10^

HF results in the activation of peripheral arterial chemoreceptors and decreased input from inhibitory baroreceptors which can lead to activation of SNS, renin-angiotensin-aldosterone (RAAS), arginine vasopressin (AVP) along with loss of parasympathetic tone and increase in resistance to natriuretic peptides.^10^ The activation of SNS, RAAS, and endothelin systems usually occurs in response to low cardiac output, worsening end-organ perfusion, and low blood pressure, contributing to ventricular remodeling and dysfunction. These lead to free-water retention and worsening of systemic vasoconstriction, further contributing to volume overload, increased ventricular afterload, and circulatory failure.^9^^,^^10^

Additionally, there is growing evidence of cross-regulation between the SNS and the innate immune system. The role of proinflammatory cytokines has been proposed in the progression of HF.^11^ Tumor necrosis factor-α (TNF-α) in particular has been shown to have negative inotropic effects by exerting oxidative stress on the cardiomyocyte, and studies have shown that overexpression of the TNF-α transgene is associated with increased mortality.^12^, ^13^, ^14^ Several studies have suggested the use of TNF-α and interleukin (IL)-6 as markers of HF.^15^^,^^16^ Other proinflammatory cytokines found to be released following cardiac stress include transforming growth factor-β (TGF-β), IL-1, IL-8, and IL-18.^16^, ^17^, ^18^

## Neuroimmune axis and the rationale of neuromodulation in HF

The neuroimmune axis is a complex pathway that interconnects the central vagal pathways, primarily in the brainstem dorsal vagal complex with the immune axis within the splenic milieu.^19^ With a complex interplay between the sympathetic and the parasympathetic neurotransmitters, norepinephrine and acetylcholine, vagal efferent activity leads to modulation of inflammation and cytokine generation within the spleen.^20^, ^21^, ^22^ Decades of work have established the relationship between inflammatory mediators such as TNF-α, IL-1 β, IL-6, C-reactive protein, etc, and HF pathophysiology. While the etiology and pathophysiological mechanisms within the various spectrums of HF are quite diverse, varying effects of these inflammatory mediators, linked to the neuroimmune axis, directly impact the progression of various HF syndromes. Modulating the neuroimmune axis in HF seems to be an attractive therapeutic target.^20^, ^21^, ^22^

The heart is innervated by preganglionic vagal motor neurons originating in the medulla oblongata and sympathetic efferent preganglionic neurons located in the spinal cord at the C8-T1 levels.^23^ In addition, the heart is believed to have its intracardiac neuronal network called the intrinsic cardiac nervous system.^23^ Neuromodulation of the autonomic nervous system aims to restore the balance between the SNS and the PNS. Given that autonomic dysregulation plays a major role in the development and progression of HF, restoring this balance may potentially have an impact on the core pathophysiological mechanisms and various HF syndromes. β-Blockers, angiotensin-converting enzyme inhibitors, sodium-glucose cotransporter-2 inhibitors, and mineralocorticoid antagonists such as spironolactone are widely used and have shown tremendous benefits in chronic HF patients with reduced ejection fraction.^24^ Several nonpharmacological therapies have been previously tested for HF. These include direct and indirect vagal nerve stimulation, spinal cord stimulation (SCS), baroreflex activation therapy (BAT), carotid sinus stimulation, aortic arch stimulation, splanchnic nerve modulation (SNM), cardiopulmonary nerve stimulation, and renal sympathetic nerve denervation (RDN) (Central Illustration). In this review, we provide a comprehensive overview of such device-based therapies for neuromodulation and HF.

**Central Illustration:**
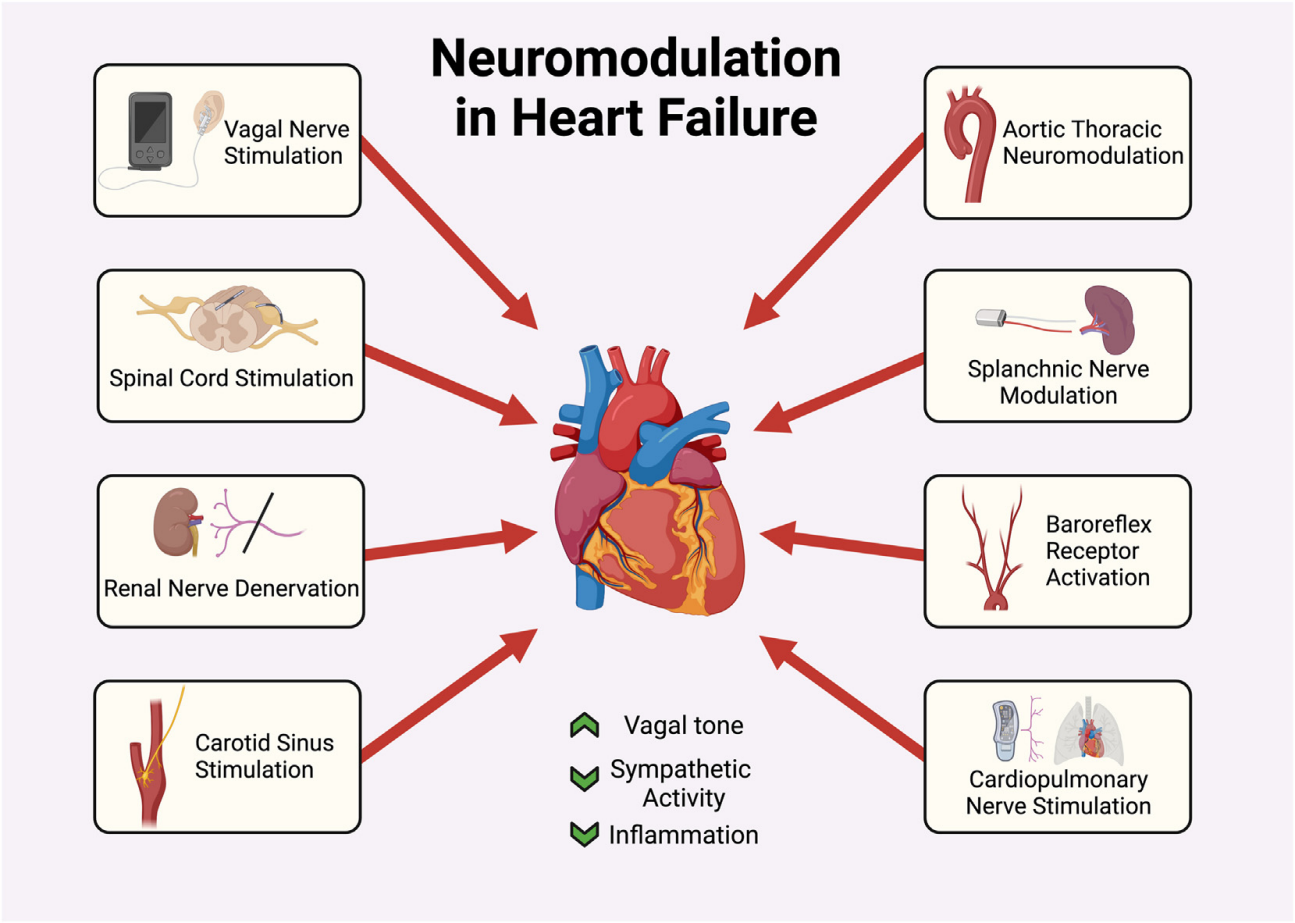
Device-based neuromodulation in heart failure.

## Current recommendations on neuromodulation in the management of HF

Most professional societies currently do not provide a clear recommendation on the use of neuromodulation devices in chronic HF patients. Most recent consensus guidelines by the American College of Cardiology and the American Heart Association released in 2022 listed the use of neuromodulation, BAT, and RDN under nonmedical strategies with evidence gaps and requiring further research.^24^ The European Society of Cardiology mentions the use of BAT as having a modest effect on the improvement of symptoms and quality of life (QOL) but with limited data on hard clinical outcomes such as mortality in patients with chronic HF.^25^

In 2019, the Barostim NEO (CVRx, Inc), a baroreceptor activation device, was granted approval by the Food and Drug Administration for use in advanced HF patients who are not candidates for other proven treatment options.^26^ This was based on the results from the Baroreflex Activation Therapy for HF (BeAT-HF) trial which showed a significant improvement in QOL measures, 6-minute walk distance (6MWD), and a reduction in N-terminal pro-B-type natriuretic peptide (NT-proBNP) level.^27^ The Society for Cardiovascular Angiography & Interventions has published a consensus statement highlighting the potential benefits of RDN for the treatment of hypertension and the possible extension of its beneficial effects on left ventricular hypertrophy.^28^^,^^29^ Currently, Barostim NEO remains the only neuromodulation device approved for use in HF.^26^

## Devices for neuromodulation, technical aspects, and evidence in HF

### Baroreflex activation therapy

The carotid body and carotid sinus are supplied both by PNS and SNS and contain both chemoreceptors and stretch-sensitive mechanoreceptors.^30^ Carotid baroreflex system modulates the autonomic balance and plays a crucial role in regulating blood pressure and heart rate.^30^ Stimulation of carotid sinus mechanoreceptors leads to downregulation of SNS.^31^ Furthermore, downregulation of SNS is associated with suppression of RAAS and muscle sympathetic nerve activity, with downstream beneficial effects on hemodynamics as evident in the reduction in systemic blood pressure.^31^^,^^32^ In a canine ischemic HF model (n = 14), bilateral BAT for 3 months was associated with reduction in left ventricular end-diastolic pressure and end-systolic volume and normalized the expression of cardiac β-1 adrenergic receptors. In addition, it also led to a reduction in myocardial interstitial fibrosis and cardiac myocyte hypertrophy.^33^

The first-generation Rheos system (CVRx) included an external programmer, an implantable pulse generator, and 2 electrodes placed at both carotid sinuses. An external programming system is used to program the implanted pulse generator via radiofrequency control.^31^^,^^34^^,^^35^ A newer generation system Barostim NEO is much less invasive and consists of a single electrode, providing unilateral stimulation. The electrode is sutured to the surface of the carotid sinus and connected to an implantable pulse generator placed in a subcutaneous pocket in the infraclavicular space.^30^^,^^31^

#### Clinical trials

The role of BAT in patients with heart HF with reduced ejection fraction (HFrEF) has been extensively studied.^36^, ^37^, ^38^ In a study by Gronda et al,^39^ 11 patients with HFrEF and New York Heart Association (NYHA) Class III symptoms received BAT for 6 months. BAT was associated with improvement in NYHA class, QOL, 6MWD, and left ventricular ejection fraction (LVEF). BAT was also associated with reduction in muscle sympathetic nerve activity (Table 1).^26^^,^^27^^,^^39^, ^41^, ^42^, ^43^, ^45^, ^46^, ^47^, ^48^, ^49^, ^50^, ^51^, ^52^, ^53^, ^54^, ^55^, ^56^, ^57^, ^58^, ^60^ The Barostim Hope for Heart Failure (HOPE4HF) study was the first large randomized controlled trial (RCT) in which 146 patients with NYHA class III HF and LVEF < 35%, on optimal medical therapy were randomized to BAT plus optimal medical therapy (n = 76) vs optimal medical therapy alone (n = 70). Results of the trial demonstrated significant improvement in 6MWD, QOL score, and NYHA functional class (Table 1).^40^ The BeAT-HF trial randomized 408 patients with NYHA class II/III HF with LVEF < 35%, on optimal medical therapy, and not meeting indication for cardiac resynchronization therapy to BAT plus medical therapy or medical therapy alone. Significant improvements in QOL score, 6MWD, and serum NT-proBNP levels were noted in the study. In this cohort, there were 245 patients who were randomized to BAT plus medical therapy (120 patients) vs medical therapy alone (125 patients). During a mean follow-up of 6 months, BAT was associated with significant improvement in 6MWD (Δ: 60 meters; 95% CI, 40-80 meters; *P* < .001), reduction in Minnesota living with heart failure QOL score (Δ: –14.1; 95% CI, –19 to –9; *P* < .001), and reduction in serum NT-proBNP levels (Δ: –25%; 95% CI, –38% to –9%; *P* = .004) (Table 1).^27^ A meta-analysis based on individual patient-level data of the Barostim HOPE4HF and BeAT-HF trials showed that benefit of BAT in patients with HFrEF is more pronounced among patients without cardiac resynchronization therapy and serum NT-proBNP < 1600 pg/mL.^26^

**Table 1:** Summary of clinical trials on device-based neuromodulation in heart failure.

Study title	Device	Study design	Patients	Key inclusion criteria	Key findings
Gronda et al^39^	BAT	Single arm, open-label	BAT: 11	LVEF < 40% NYHA: III	BAT showed improvement in MSNA, LVEF, NYHA class, QOL, and 6MWD
Barostim HOPE4HF^26^^,^^40^	BAT	Open-label, RCT	BAT: 76 Control: 70	LVEF < 35% NYHA: III	BAT showed improvement in 6MWD, QOL, NYHA class, and reduced serum NT-proBNP
BeAT-HF^27^	BAT	Open-label, RCT	BAT: 120 Control: 125	LVEF < 35% NYHA: II-III	BAT showed improvement in 6MWD and QOL, and reduced serum NT-proBNP
Ferrari et al^41^	VNS	Single arm, open-label	VNS: 32	LVEF < 35% NYHA: II-IV	VNS showed improvement in 6MWD, NYHA class, LVEF, and LV systolic volume
NECTAR-HF^42^	VNS	RCT	VNS: 63 Control: 32	LVEF < 35% NYHA: II-III	VNS showed improvement in QOL and NYHA class; no significant difference in LVESD, LVESV, LVEF, and serum NT-proBNP
ANTHEM-HF^43^^,^^44^	VNS	Open-label, RCT	Right VNS: 29 Left VNS: 31	LVEF < 40% NYHA: II-III	VNS showed improvement in LVEF, LVESV, 6MWD, and NYHA class; no significant difference between right vs left VNS
INOVATE-HF^45^	VNS	Open-label, RCT	VNS: 436 Control: 271	LVEF < 40% NYHA: III	VNS showed improvement in QOL, NYHA class, and 6MWD; no significant difference was observed in mortality or HF events
ANTHEM-HFpEF^46^	VNS	Single arm, open-label	VNS: 52	LVEF > 40% NYHA: II-III	VNS showed improvement in QOL, NYHA class, HRV, and 6MWD; no difference in echocardiographic parameters of E/e' ratio, LVEF, LVESV, and LV mass index
Stavrakis et al^47^	LLTS	RCT	LLTS: 24 Control: 24	LVEF > 50% BNP > 35pg/mL	VNS through LLTS was associated with improvement in QOL, global longitudinal strain, and systemic inflammation
SCS-HEART^48^	SCS	Open-label, pilot trial	SCS: 17 Control: 4	LVEF 20-35% NYHA: III	SCS showed improvement in NYHA class, QOL, LVEF, and LVESV; no significant difference in serum NT-proBNP
DEFEAT-HF^49^	SCS	RCT	SCS: 42 Control: 24	LVEF < 35% NYHA: III	SCS showed no difference in LVESVi, QOL, 6MWD, NYHA class; no difference in all-cause mortality or hospitalization for HF
REACH^50^	RDN	Open-label, pilot trial	RDN: 7	LVEF < 45% NYHA: III-IV	RDN showed improvement In QOL and 6MWD
Symplicity HF^51^	RDN	Single arm, open-label	RDN: 39	LVEF < 40% NYHA: II-III	RDN showed reduction in serum NT-proBNP; no improvement in 6MWD, LVEF, and renal function
Chen et al^52^	RDN	Open-label, RCT	RDN: 30 Control: 30	LVEF < 40% NYHA: II-IV	RDN showed improvement in serum NT-proBNP, 6MWD, NYHA class, and LVEF; no significant difference in renal function
Gao et al^53^	RDN	Open-label, RCT	RDN: 30 Control: 30	LVEF < 40% NYHA: II-III	RDN showed improvement in serum NT-proBNP, NYHA class, LVEF, and BP; no difference in renal function and heart rate
RDT-PEF^54^	RDN	Open-label, RCT	RDN: 17 Control: 8	LVEF > 50% NYHA: II-III	RDN showed no difference in QOL, peak oxygen uptake, serum BNP, left atrial volume index, and LV mass index
HF-FIM^55^	EVBA	Single arm, open-label	EVBA: 29	LVEF < 40% NYHA: II-III	EVBA showed improvement in QOL, 6MWD, LVEF, and serum NT-proBNP
Splanchnic HF-1^56^	SNM	Single arm, open-label	SNM: 11	LVEF < 45% NYHA: III-IV	SNM showed improvement in PCWP, and left atrial volume index
Splanchnic HF-2^57^	SNM	Single arm, open-label	SNM: 15	LVEF < 35% NYHA: II-III	SNM showed improvement in resting PCWP; improvement in peak-exercise PCWP, mean PA pressure, and cardiac index
Málek et al^58^	SNM	Single arm, open-label	SNM: 10	LVEF > 40% NYHA: III	SNM showed improvement in peak-exercise PCWP, QOL, and NYHA class
Fudim et al^59^	SNM	Single arm, open-label	SNM: 11	LVEF > 50% NYHA: II-III	SNM showed improvement in 6MWD, QOL, and serum NT-proBNP
Reddy et al^60^	CPNS	Single arm, open-label	CPNS: 10	LVEF < 35%	CPNS showed improvement in LV contractility and arterial pressure; no change in heart rate or LV filling pressures

6MWD, 6-minute walk distance; ANTHEM-HF, Autonomic Neural Regulation Therapy of Enhance Myocardial Function in Heart Failure; ANTHEM-HFpEF, Autonomic Neural Regulation Therapy to Enhance Myocardial Function in Heart Failure with Preserved Ejection Fraction; BAT, baroreflex activation therapy; BeAT-HF, Baroreflex Activation Therapy for Heart Failure; BP, blood pressure; CPNS, cardiac pulmonary nerve stimulation; DEFEAT-HF, Determining the Feasibility of Spinal Cord Neuromodulation for the Treatment of Chronic Heart Failure; EVBA, endovascular baroreflex activation; HF, heart failure; HF-FIM, MobiusHD Device in Patients with Heart Failure; HOPE4HF, Hope for Heart Failure; HRV, heart rate variability; INOVATE-HF, Increase of Vagal Tone in Congestive Heart Failure; LLTS, low-level tragus stimulation; LV, left ventricle; LVEF, left ventricular ejection fraction; LVESD, left ventricular end-systolic diameter; LVESV, left ventricular end-systolic volume; LVESVi, left ventricular end-systolic volume index; MSNA, muscle sympathetic nerve activity; NECTAR-HF, Neural Cardiac Therapy for Heart Failure; NT-proBNP, N-terminal pro-brain natriuretic peptide; NYHA, New York Heart Association functional classification; PA, pulmonary artery; PCWP, pulmonary capillary wedge pressure; QOL, quality of life; RCT, randomized controlled trial; RDN, renal sympathetic nerve denervation; RDT-PEF, Renal Denervation in Heart Failure with Preserved Ejection Fraction; REACH, Renal Artery Denervation in Chronic Heart Failure; SCS, spinal cord stimulation; SCS-HEART, Spinal Cord Stimulation for Heart Failure; SNM, splanchnic nerve modulation; Symplicity-HF, Sympathetic Response and Outcomes Following Renal Denervation in Patients with Chronic Heart Failure; Splanchnic HF-1, Splanchnic Nerve Block for Decompensated Chronic Heart Failure; Splanchnic HF-2, Abdominal Nerve Blockade in Chronic Heart Failure; VNS, vagus nerve stimulation.

#### Ongoing trials

The Rheos Diastolic Heart Failure Trial (NCT00718939),^61^ and Rheos System for the Treatment of Heart Failure With Preserved Ejection Fraction (HFpEF) Heart Failure (HOPE4HF) trial (NCT00957073)^62^ are currently ongoing (Table 2).^63^, ^64^, ^65^, ^66^, ^67^, ^68^, ^69^, ^70^, ^71^, ^72^ Both these trials aim to evaluate the safety and efficacy of BAT in patients with HF with preserved ejection fraction (LVEF > 40%). End points include safety, major adverse cardiovascular events, changes in blood pressure, serum levels of biomarkers, QOL, and left ventricular mass index. Although trials have shown potential benefit in patients with HFrEF, there is limited evidence in terms of reduction in mortality or hospitalizations for HF.

**Table 2:** Summary of ongoing studies on device-based neuromodulation in heart failure.

Study title	Device	Study design	N	Key inclusion criteria	Outcomes of interest
ANTHEM-HFrEF NCT03425422^63^	VNS	Open-label, RCT	533	LVEF < 35% NYHA: II-III LVEDD < 8.0 cm	Device implantation-related serious adverse events, composite of CV mortality and hospitalization for HF
RE-ADAPT-HF NCT04947670^64^	RDN	RCT	144	LVEF < 45% NYHA: II-III 6MWD < 350 m	Changes in 6MWD, QOL score, renal function, and serum NT-proBNP levels at 6 mo
UNLOAD-HFpEF NCT05030987^65^	RDN	RCT	68	LVEF > 55% NYHA: II-III LVEDP > 16 mm Hg PCWP > 15 mm Hg	Exercise PCWP at 6 mo, change in QOL score, 6MWD, reduction in mortality and hospitalization for HF, and difference in serum NT-proBNP levels
DIASTOLE NCT01583881^66^	RDN	Open-label, RCT	60	LVEF > 50% SBP > 140 mm Hg DBP > 90 mm Hg	Change in E/E' on echocardiography at 12 mo, change in LV mass, LV volume, LVEF, and left atrial volume
ENDO-HF NCT02633644^67^	ATN	Single arm, open-label	30	NYHA: II-III	Procedure-related serious adverse events, change in NYHA class, 6MWD, QOL score, LVEF, LVESV, LVEDV, and LV mass index at 6 mo
REBALANCE-HF NCT04592445^68^	SNM	RCT	80	LVEF > 50% NYHA: II-IV >1 Hospitalization for HF PCWP ≥ 25 mm Hg during exercise	Change in PCWP at 1 m, procedure-related serious adverse events, change in QOL score, 6MWD, incidence of hospitalization for HF at 12 mo
Cardionomic STOP-ADHF NCT04814134^69^	CPNS	Open-label, nonrandomized	90	Hospitalization for ADHF LVEF < 50% NT-proBNP > 2000 pg/mL	Device or procedure-related serious adverse events, all-cause mortality
TREAT-HF NCT02898181^70^	LLTS	RCT	80	LVEF < 40% Hospitalization for ADHF	Change in C-reactive protein, TNF-α, and interleukin levels
TRANSFER NCT05789147^71^	LLTS	Open-label, RCT	40	NYHA: II-III	Heart rate and baroreflex sensitivity
Neuromodulation of Inflammation and Endothelial Function NCT05230732^72^	LLTS	RCT	158	LVEF < 40%	Change in 6MWD, QOL scores, HRV, C-reactive protein, NT-proBNP, TNF-α, and interleukin levels

6MWD, 6-minute walk distance; ADHF, acute decompensated heart failure; ANTHEM-HFrEF, Autonomic Regulation Therapy to Enhance Myocardial Function and Reduce Progression of Heart Failure With Reduced Ejection Fraction; ATN, aortic thoracic neuromodulation; Cardionomic STOP-ADHF, Stimulation of the Cardiopulmonary Nervous System in Acute Decompensated Heart Failure; CPNS, cardiac pulmonary nerve stimulation; CV, cardiovascular; DBP, diastolic blood pressure; DIASTOLE, Denervation of the Renal Sympathetic Nerves in Heart Failure with Normal LV Ejection Fraction; ENDO-HF, Endovascular NeuromoDulation for Heart Failure; HF, heart failure; HRV, heart rate variability; LLTS, low-level tragus stimulation; LV, left ventricle; LVEDD, left ventricular end-diastolic diameter; LVEDP, left ventricular end-diastolic pressure; LVEDV, left ventricular end-diastolic volume; LVEF, left ventricular ejection fraction; LVESV, left ventricular end-systolic volume; N, number; N, number of patients; NT-proBNP, N-terminal pro-brain natriuretic peptide; NYHA, New York Heart Association functional classification; PCWP, pulmonary capillary wedge pressure; QOL, quality of life; RCT, randomized controlled trial; RDN, renal sympathetic nerve denervation; RE-ADAPT-HF, A Prospective, Multicenter, Randomized, Blinded, Sham-controlled, Feasibility Study of Renal Denervation in Patients with Chronic ​Heart ​Failure; REBALANCE-HF, Endovascular Ablation of the Right Greater Splanchnic Nerve in Subjects Having HFpEF; SBP, systolic blood pressure; SCS, spinal cord stimulation; SNM, splanchnic nerve modulation; TNF-α, tumor necrosis factor-α; TRANSFER, Transcutaneous ​Vagus Nerve Stimulation ​in ​Heart Failure; TREAT-HF, Low-Level Tragus Stimulation in Acute Decompensated Heart Failure; UNLOAD-HFpEF, Renal Denervation to Treat ​Heart ​Failure ​with Preserved Ejection Fraction; VNS, vagus nerve stimulation.

### Vagus nerve stimulation

Vagus nerve stimulation (VNS) aims to restore autonomic balance by upregulating vagal activity.^73^ VNS has been successfully used to treat refractory depression and epilepsy.^74^^,^^75^ Increased sympathetic activity is associated with worse outcomes in patients with HF.^37^ Animal models of HF have shown that autonomic modulation via VNS is associated with improvement in hemodynamics, reduction in plasma norepinephrine, restoration of baroreflex sensitivity, and improved survival.^76^^,^^77^ VNS has a favorable effect on systemic inflammation by reducing proinflammatory cytokines.^76^^,^^78^ VNS has been shown to augment nitric oxide levels in cardiac myocytes, leading to increased ventricular contractility.^30^^,^^79^ VNS also leads to significant reduction in SNS activity from left stellate ganglion and reduces the frequency of atrial arrhythmias in patients with HF.^47^^,^^80^

#### Clinical trials

Ferrari et al^41^ performed an initial open-label, phase II trial using the CardioFit VNS system (BioControl Medical) in 32 patients with symptomatic NYHA class II to IV HF (LVEF < 35%). Right closed loop cervical VNS (preferentially efferent stimulation to maximize B-type fiber recruitment while minimizing A-type fiber recruitment) was performed to assess tolerability and safety at 1 year. No major safety concerns were noted in the study (Table 1). Autonomic modulation via VNS has been evaluated in 3 pivotal clinical trials: Autonomic Neural Regulation Therapy of Enhance Myocardial Function in Heart Failure (ANTHEM-HF), Neural Cardiac Therapy for Heart Failure, and Increase of Vagal Tone in Congestive Heart Failure (INOVATE-HF).^42^, ^43^, ^45^

The ANTHEM-HF was an open-label phase II trial in 60 patients with NYHA Class II to III HF (LVEF < 40%). Patients were randomized in 1:1 fashion to undergo either right or left cervical vagal nerve stimulation using a bipolar vagal electrode without preferential efferent stimulation at 10 Hz frequency, without electrocardiogram synchronization, with a mean output current of 2 ± 0.6 mA (PerenniaFLEX Model 304 lead, Cyberonics).^43^ In the study, pooled analysis revealed a slightly favorable profile with right VNS with an increase in LVEF, QoL, and NYHA symptoms.^43^ Improvement in symptoms and QOL are maintained at 12 and 42 months with no differences between right and left-sided VNS (Table 1).^44^

Neural Cardiac Therapy for Heart Failure was the first phase II trial evaluating right-sided VNS in patients with HFrEF. The trial randomized 96 patients with NYHA Class II to III HF, and LVEF < 35%, on stable medical therapy. All patients underwent implantation and were subsequently randomized in a 2-1 fashion to VNS using bipolar vagal electrode without preferential efferent stimulation vs sham for the first 6 months and subsequently VNS was turned on and all patients. At a follow-up period of 6 months, VNS at 20 Hz was not associated with significant changes in left ventricular volumes, ejection fraction, or NT-proBNP but led to an improvement in QOL and NYHA functional class. At the 18-month follow-up, only the NYHA class remained significantly different between the 2 groups. No significant changes were noted in the heart rate variability (HRV)-time frequency domain measures during the follow-up (Table 1).^42^

The INOVATE-HF phase III trial was a large multicenter RCT evaluating the efficacy of VNS in patients with chronic HF.^45^ The study enrolled 707 patients with symptomatic NYHA functional Class III HF with reduced LVEF (<40%). The trial randomized patients in a 3:2 fashion to receive VNS vs continuation of optimal medical therapy. CardioFit VNS system (BioControl Medical) was utilized to provide only right-sided VNS and an intracardiac right ventricular sensing lead. Stimulation output was gradually increased to achieve a target current of 3.5 to 5.5 mA. The primary efficacy end point was a composite of death or HF-related hospitalization. During a mean follow-up period of 16 months, no statistically significant difference was observed in the primary end point of death or HF-related hospitalization but secondary end points including ventricular volumes, QOL, NYHA class, and 6MWD improved by VNS (the study was unblinded) (Table 1).^45^

The different stimulation parameters used in VNS studies may account for the heterogeneity in the results.^81^ Therefore, identifying optimal stimulation parameters is crucial to the success of neuromodulation therapies.^82^ Likewise, patient selection is equally important to optimize neuromodulation outcomes.^81^^,^^82^ Unfortunately, an accurate biomarker of response to neuromodulation therapy, able to guide therapy, is lacking at present. A few studies have used the effect on heart rate and/or HRV as a surrogate marker for the cardiac effects of VNS.^83^^,^^84^ It should be noted, however, that heart rate is determined by mixed inputs from the SNS and PNS, and therefore the heart rate-lowering effect of VNS may not necessarily correlate with the effects on cardiac function.^82^ Similarly, the use of HRV is limited by the fact that changes in HRV are correlated with the effect of the autonomic nervous system on the sinus node and not necessarily with the outcome of interest.^82^ Further research is required to identify biomarkers of response to neuromodulation therapy to guide patient selection and optimize the results of neuromodulation therapies.

Autonomic Neural Regulation Therapy to Enhance Myocardial Function in HF with Preserved Ejection Fraction study (NCT03163030) was the first trial to assess safety and feasibility of VNS in patients with HFpEF and HF with mildly reduced ejection fraction (HFmrEF).^46^, ^85^ The trial enrolled 52 patients with HFpEF and HFmrEF, NYHA Class II to III, and LVEF > 40% to undergo right cervical VNS via the LivaNova VNS Therapy system (LivaNova). Trial results showed VNS was safe, and was associated with improvement in QOL, NYHA class, and 6MWD at 12 months of follow-up. VNS showed improvement in HRV and reduced incidence of nonsustained ventricular tachycardia. There was no difference in echocardiographic parameters of E/e' ratio, LVEF, left ventricular end-systolic volume (LVESV) and left ventricle (LV) mass index (Table 1).^46^

#### Ongoing trials

Autonomic Regulation Therapy to Enhance Myocardial Function and Reduce Progression of HF With Reduced Ejection Fraction Pivotal study (NCT03425422) is currently ongoing (Table 2).^63^^,^^86^ The study will randomize patients to autonomic regulation therapy vs medical therapy alone with a primary efficacy end point of composite of cardiovascular death or first hospitalization for HF.^63^^,^^86^

## Spinal cord stimulation

Spinal cord stimulation has been utilized effectively in the management of chronic pain and angina.^87^^,^^88^ The procedure involves percutaneous placement of a multipolar lead into the epidural space under fluoroscopic guidance.^89^^,^^90^ An implanted pulse generator placed in the paraspinal lumbar region is connected to the electrode. SCS is performed typically at 90% of the motor threshold, with a frequency of 50 Hz and pulse width of 200 milliseconds.^89^ SCS has been shown to upregulate the activity of PNS through enhanced vagal tone, potentially leading to a benefit in patients with HF.^91^ Canine models of HF have shown that SCS leads to a reduction in ventricular arrhythmias and improvement in LV systolic function.^92^^,^^93^ Similar benefit in terms of reduction in infarct size and ventricular arrhythmias were observed with SCS in porcine models of ischemia-reperfusion injury.^92^^,^^94^

### Clinical trials

Spinal Cord Stimulation for Heart Failure (SCS-HEART) was the first pilot study evaluating the role of SCS in patients with HF.^48^ The trial enrolled 22 patients with symptomatic HF (NYHA Class III) with reduced LVEF (LVEF 20%-35%), on stable medical therapy with implantable cardioverter defibrillator. Seventeen patients underwent percutaneous placement of dual thoracic SCS leads at higher thoracic level (T1-T3). Patients received continuous SCS for 24 hours per day, at a frequency of 50 Hz and pulse width of 200 μs. Four patients who did not fulfill the study criteria served as nontreated controls. At a follow-up period of 6 months, SCS showed significant improvement in QOL scores, NYHA class, and LVESV in 17 patients who were treated with SCS compared with 4 nontreated patients. Furthermore, LVEF was favorably affected in patients receiving SCS at follow-up (25% vs 37%, *P* < .001). SCS did not show improvement in serum NT pro-BNP levels (Table 1).^48^

Encouraging results of the SCS-HEART study led to further investigation into the role of SCS in chronic HF. Determining the Feasibility of Spinal Cord Neuromodulation for the Treatment of Chronic Heart Failure (DEFEAT-HF) study was a multicenter RCT that enrolled 81 patients with NYHA Class III HF, LVEF < 35%, QRS duration <120 milliseconds, and LV end-diastolic dimension >55 mm.^49^ A total of 66 patients received SCS and were randomized in 3:2 fashion to active therapy (SCS ON), vs control (SCS OFF). SCS therapy was accomplished by a single lead with 8 electrodes, implanted at T2 to T4 levels. Patients received SCS for 12 hours per day, at a frequency of 50 Hz and pulse width of 200 μs. During a follow-up duration of 6 months, the primary outcome of the LV end-systolic volume index did not show a significant difference between both the groups. Secondary outcomes including QOL scores, 6MWD, and change in NYHA class did not show a difference between both the groups. Furthermore, SCS did not show a significant reduction in hospitalization for HF or all-cause mortality at 6 months (Table 1).^49^ Discordance in the results of SCS-HEART and DEFEAT-HF could be secondary to different stimulation protocols utilized in both the trials. SCS-HEART utilized dual thoracic SCS leads at higher thoracic levels (T1-T3), whereas DEFEAT-HF used a single lead with 8 electrodes, implanted at T2 to T4 levels. Furthermore, patients in SCS-HEART received continuous SCS for 24 hours per day, whereas patients in DEFEAT-HF received SCS for 12 hours per day.^48^^,^^49^ Preclinical studies have shown that SCS delivered at the T4 level was associated with more antiarrhythmic activity, whereas SCS at the T1 level leads to upregulation of PNS via a higher vagal tone.^92^ Whether or not differences in stimulation techniques and protocol could have led to differences in outcomes, needs to be evaluated further in future trials. However, enrollment of patients in trials evaluating SCS in HF is logistically challenging. In fact, A Pilot Study of Spinal Cord Stimulation in Heart Failure Patients with Depressed Left Ventricular Function trial (NCT02450110) had to be terminated due to slow recruitment rate.^95^

### Renal denervation

Autonomic modulation via RDN has received increased attention in recent years. Dysfunction in the autonomic nervous system and RAAS are pathologically responsible for worse clinical outcomes in patients with HF.^96^ Reduced cardiac output and resulting diminished renal perfusion leads to activation of RAAS, which subsequently leads to increased central SNS output.^97^ Furthermore, increased SNS activity in patients with HF can itself lead to increased RAAS activity.^98^^,^^99^ Dysfunction in the bidirectional feedback between SNS and RAAS leads to pathological left ventricular remodeling, abnormal fluid and sodium retention, and increased peripheral vascular resistance, ultimately leading to increase in morbidity and mortality in patients with HF.^30^ Although initially evaluated in patients with resistant hypertension, theoretically, RDN appears to be a promising therapy in patients with HF.^100^

Low-energy radiofrequency ablation catheter and denervation are performed by circumferential application within the renal artery. In general, 4 to 8 applications are applied along the length of the renal artery all the way up to the second-order renal artery branches. Potential complications include renal artery perforation and renal dissection.^30^^,^^101^ Recent advances in the technique include utilization of different energy sources (cryoablation, laser, ultrasound), and ablation catheter designs (unipolar, bipolar, multipolar).^30^^,^^101^ Animal models of HF have demonstrated reduction in angiotensin receptor density and improvement in cardiac output following RDN.^102^

#### Clinical trials

The Renal Artery Denervation in Chronic Heart Failure trial was the first human study evaluating safety and efficacy of RDN in patients with HF.^50^ The study enrolled 7 patients with chronic systolic HF who received bilateral RDN. RDN was safe with no postprocedure complications, and was well-tolerated with no episodes of hypotension or syncope, or worsening of renal function at 6 months. Patients experienced improvement in QOL along with improved 6MWD (Table 1).^50^ Sympathetic Response and Outcomes Following Renal Denervation in Patients with Chronic HF study was another study that enrolled 39 patients with symptomatic HF and LVEF < 40%.^51^ RDN therapy was associated with significant reduction in serum NT-proBNP levels. No significant improvement was observed in 6MWD, LVEF, or renal function among patients (Table 1).^51^

Chen et al^52^ performed an RCT enrolling 60 patients with symptomatic systolic HF. Patients were randomized to RDN (30 patients) vs control (30 patients). During a mean follow-up of 6 months, patients receiving RDN experienced significant improvements in 6MWD, NYHA class, LVEF, and serum NT-proBNP levels. No significant difference was observed in renal function between both groups (Table 1). Another RCT by Gao et al^53^ evaluated the effects of RDN in patients with systolic HF. The trial randomized 60 patients with chronic symptomatic HF to RDN vs control. During a mean follow-up of 6 months, RDN was associated with a significant reduction in serum NT-proBNP levels as compared with control. Furthermore, NYHA functional class and blood pressure were favorably affected by RDN. Patients receiving RDN also showed significant improvement in LVEF as compared with control (39.1% vs 35.6%, *P* = .017) (Table 1).

RDN in patients with HF with preserved ejection fraction (HFpEF) has received recent interest. The Renal Denervation in HF with Preserved Ejection Fraction trial recruited 25 patients with HFpEF. Patients were randomized in a 2:1 fashion to RDN vs control. The study struggled with recruitment of patients and during a follow-up period of 12 months, no difference was observed in terms of QOL score, peak oxygen uptake (VO_2_) on exercise, serum brain natriuretic peptide levels, and echocardiographic assessment of left atrial volume index or left ventricular mass index (Table 1).^54^

#### Ongoing trials

A Prospective, Multicenter, Randomized, Blinded, Sham-controlled, Feasibility Study of Renal Denervation in Patients with Chronic Heart Failure trial (NCT04947670) is currently ongoing and plans to recruit 144 patients with symptomatic HF and reduced LVEF (<45%). The study will evaluate the effect of RDN on QOL and functional capacity in patients with HFrEF (Table 2).^64^ Renal Denervation to Treat Heart Failure with Preserved Ejection Fraction trial (NCT05030987) is currently enrolling patients to undergo RDN in comparison with sham-control. Efficacy end points include effects of RDN of pulmonary capillary wedge pressure during exercise, QOL scores, 6MWD, reduction in mortality and hospitalization for HF, and difference in serum NT-proBNP levels (Table 2).^65^ Denervation of the Renal Sympathetic Nerves in Heart Failure with Normal LV Ejection Fraction trial (NCT01583881) will randomize 60 patients with HFpEF to RDN vs optimal medical therapy. Primary outcome of interest is improvement in Doppler echocardiographic parameters. Key secondary outcomes include change in LV mass, LV volume, LVEF, and left atrial volume as determined by magnetic resonance imaging (Table 2).^66^

Trials to date have demonstrated meaningful improvements in QOL and functional capacity with RDN. However, the effects on hard clinical end points are yet to be evaluated in larger clinical trials. This however might be logistically challenging due to rates of low enrollment. Nonetheless, renal nerve denervation therapy may continue to hold promise and further research is warranted to better understand the underlying mechanisms driving the clinical benefit in patients with systolic HF.

### Endovascular baroreflex activation

Endovascular baroreflex activation (EVBA) is a promising therapy, designed to reduce SNS over activity. The self-expanding MobiusHD device (Vascular Dynamics) is a nitinol self-expanding stent implanted in internal carotid artery. EVBA, via this device, changes the geometric shape of the carotid sinus and increases pulsatile wall strain. EVBA is aimed to augment baroreflex mechanism and carotid baroreceptor signaling, ultimately leading to downregulation of SNS activity in patients with HF.^55^ EVBA has shown promising results in patients with resistant hypertension.^103^^,^^104^ Upregulation of PNS and downregulation of SNS via baroreflex activation could likely lead to improvement in functional status in patients with HF.

#### Clinical trial

Mobius HD Device in Patients with Heart Failure study (NCT04590001) is a pilot study evaluating the effect of EVBA in patients with HF (Table 1).^55^ The study enrolled 29 patients with symptomatic HF (NYHA class II-III), with LVEF < 40%, and serum NT-proBNP > 400 pg/mL. The MobiusHD device placement was successful in all patients. During a follow-up period of 12 months, favorable improvement in QOL scores, 6MWD, and LVEF were observed among patients undergoing EVBA. Furthermore, EVBA was associated with a mean reduction of 28% in serum levels of NT-proBNP at follow-up (Table 1).^55^ Encouraging results of the pilot study call for further RCT with adequate power to evaluate the efficacy of EVBA in this vulnerable patient population.

## Aortic thoracic neuromodulation

Thoracic aortic vagal afferent fibers mediate parasympathetic signaling. In addition, aortic endothelial cells control aortic compliance and participate to restore autonomic balance.^105^^,^^106^ Chronic stimulation of aortic vagal afferent fibers is a novel interventional therapy designed to modulate the autonomic nervous system in patients with HF. The Harmony System (Enopace) consists of an implantable unit, a delivery catheter, and a programming unit. The implantable unit is a nitinol stent implant with 4 stimulation electrodes, a radiofrequency antennal coil, and a sealed electrical circuit unit. A wearable patient unit can be programmed by a physician to set the therapeutic intensity and duty cycle. Therapeutic intensity and duty cycle can be optimized via the programmable unit.^105^

Experience with aortic thoracic neuromodulation is limited. Preliminary studies have demonstrated safety and improvement in functional capacity and echocardiographic parameters. The Endovascular NeuromoDulation for Heart Failure study (NCT02633644) is currently ongoing and plans to investigate the safety and efficacy of aortic thoracic neuromodulation in patients with HF.^67^^,^^105^ The trial plans to enroll 30 patients with symptomatic HF on stable medical therapy to undergo placement of The Harmony System. End point measures include assessment of safety, QOL scores, 6MWD, NYHA class, and echocardiographic parameters including LVEF, LVESV, and LV mass index (Table 2).^67^

## Splanchnic nerve modulation

The splanchnic nerves provide autonomic innervation to the upper abdominal viscera, and SNS-mediated splanchnic vasoconstriction causes a shift of blood from the splanchnic compartment to the heart and lungs, which is a normal physiological response during exercise.^73^^,^^107^ In patients with HF, rapid shift in volume from the vascular bed to central vasculature can lead to elevated intracardiac filling pressures. Theoretically, SNM could prevent splanchnic vasoconstriction and subsequently lead to reduction in redistribution of volume from vascular beds to central vasculature.^73^ SNM can be accomplished by unilateral or bilateral chemical, electrical, or surgical greater splanchnic nerve (GSN) blockage.^73^^,^^107^ Radiofrequency ablation of GSN can be performed by a transvenous procedure (Axon Therapies). Using femoral venous access, right azygous vein and branching intercostal veins can be accessed. Right GSN and right splanchnic vein cross at the 10th and 11th thoracic levels. Under direct fluoroscopic guidance, radiofrequency energy is delivered by the ablation catheter to perform GSN blockage.^108^

### Clinical trials

Splanchnic Nerve Block for Decompensated Chronic Heart Failure study (NCT02669407) was a proof-of-concept study in 11 patients with HFrEF hospitalized for management of acute decompensated HF.^56^ Bilateral temporary splanchnic nerve blockage with needle-based injection of lidocaine was performed. Study results showed reduction in mean pulmonary capillary wedge pressure (PCWP) from 30 ± 7 mm Hg at baseline to 22 ±7 mm Hg at 30 minutes (*P* < .001). SNM also showed reduction in mean left atrial volume index following SNB (76 ± 23 mL vs 64 ± 12 mL; *P* = .043) (Table 1).^56^ Abdominal Nerve Blockade in Chronic HF study (NCT03453151) enrolled 15 patients with chronic HF. Majority of the patients (93%) had LVEF < 35%, and all patients received splanchnic nerve blockage with ropivacaine. Splanchnic nerve blockage reduced resting PCWP from 28.3 ± 7.6 mm Hg to 20.3 ± 9.5 mm Hg (*P* < .001), and peak-exercise PCWP from 34.8 ± 10 mm Hg to 25.1 ± 10.7 mm Hg (*P* < .001).^57^ The study results also showed improvement in cardiac index at peak exercise and peak oxygen consumption (Table 1).^57^

Surgical Resection of the Greater Splanchnic Nerve in Subjects Having HFpEF (NCT03715543) evaluated the safety and efficacy of permanent surgical ablation of right GSN in patients with HFpEF. In this single-arm study, 10 patients with NYHA Class III HF and LVEF > 40%, with PCWP ≥15 mm Hg at rest or ≥25 mm Hg with supine cycle ergometry, underwent ablation of the right GSN via thoracoscopic surgery.^58^ Trial results showed permanent GSN was safe, and showed favorable reduction in PCWP at 3 months, along with improvement in NYHA class and QOL scores at 12 months (Table 1).^58^ Endovascular GSN Ablation in Subjects With HFpEF study (NCT04287946) was the first human study to evaluate safety and efficacy of transvenous GSN in patients with HF.^59^ The study enrolled 11 patients with NYHA class II/III symptoms, LVEF ≥ 50%, and elevated PCWP. All patients underwent transvenous right GSN ablation using RF catheter (Axon Therapies). The study results at a median follow-up of 12 months showed that transvenous GSN ablation was safe, and showed improvement in 6MWD and QOL scores, along with favorable reduction in serum NT-proBNP levels (Table 1).^59^

### Ongoing trials

Endovascular Ablation of the Right Greater Splanchnic Nerve in Subjects Having HFpEF trial (NCT04592445) is the first sham-controlled trial assessing safety and efficacy of SNM in patients with HFpEF.^109^^,^^68^ The open-label (roll-in) arm of the Right Greater Splanchnic Nerve in Subjects Having HFpEF trial will enroll up to 30 patients, followed by the randomized, sham-controlled portion of the trial (up to 80 additional patients). The trial is currently enrolling patients with symptomatic HF, LVEF ≥50%, and PCWP ≥25 mm Hg to receive transvenous GSN ablation vs sham-control. Study outcomes include change in PCWP and safety of endovascular GSN ablation. Secondary key outcomes include changes in QOL score, 6MWD, NYHA class, and serum NT-proBNP levels (Table 2).^109^ Preliminary results from 18 patients included in the open-label (roll-in) arm show improvement in the PCWP, QOL scores, and NYHA class.^109^ Results of the ongoing sham-control study are awaited and will provide evidence on the safety and efficacy of this novel therapy in patients with HF.

## Cardiac pulmonary nerve stimulation

The cardiovascular system is richly innervated by PNS and SNS. Autonomic fibers regulating inotropic and lusitropic states of ventricles pass along the anterior and posterior surfaces of the right and main pulmonary arteries.^110^ This anatomic relation provides the basis for a novel catheter-based therapy aimed at directly modulating autonomic activity of the heart. The Cardiac Pulmonary Nerve Stimulation (CPNS) System (Cardionomic, Inc) is a catheter-based device that can directly stimulate the autonomic nerves surrounding the right pulmonary artery (RPA).^110^ The CPNS system consists of a neuromodulation stimulation catheter (CN2 catheter) with a 16-electrode nitinol braid, a stimulator that delivers electrical stimulation via the CN2 catheter, and an electrocardiogram denoiser that receives electrocardiogram signals from the patient and delivers to the patient monitor and stimulator. CN2 catheter is delivered percutaneously under direct fluoroscopic guidance and is deployed in RPA to provide electrical stimulation. The system can remain in place to provide stimulation for up to 5 days and can be retrieved after completion of therapy.^110^ Canine studies have shown that CPNS was associated with improvement in left ventricular contractility.^111^

Reddy et al^60^ performed the first human proof-of-concept study, in which 15 patients with HFrEF underwent stimulation of cardiac pulmonary nerve via RPA. Study results showed improvement in LV contractility and arterial pressure, without a significant change in heart rate or LV filling pressures (Table 1). The Stimulation of the Cardiopulmonary Nervous System in Acute Decompensated Heart Failure (Cardionomic STOP-ADHF) Study (NCT04814134) is currently ongoing and aims to understand the safety and efficacy of CPNS in patients admitted for management of acute decompensated HF.^69^ The trial plans to enroll up to 90 patients with LVEF < 50% admitted in the setting of exacerbation of HF. All patients will undergo acute ≤5 days of endovascular stimulation of the cardiac autonomic nerves in the RPA (Table 2). This trial will provide further evidence on safety and efficacy of CPNS in patients with HF.

## Unmet clinical need and evolving technologies

Despite major advances in our understanding of pathophysiology of HF and targeted device and medical therapy for HF, mortality and morbidity remains high among patients with HF. Although lacking survival benefits, BAT is still the only neuromodulation device approved for use in HF by the Food and Drug Administration.^112^ There have been several newer drug therapies such as angiotensin receptor neprilysin inhibitors and sodium-glucose cotransporter-2 (SGLT2) inhibitors, which have shown significant improvement in mortality and hospitalization. They are increasingly being utilized in management of both HFrEF and HFpEF patients. However, many of these drugs still continue to have a significant copay, and patients suffer large out-of-pocket health care expenses.^113^ Utilizing targeted devices for neuromodulation therapies provides financial benefit to the patients as it decreases their monthly expenditure for medications. Device-based neuromodulation of HF is particularly challenging in terms of finding the appropriate dosing parameters associated with clinical benefit. Furthermore, the majority of the trials in the field have been limited by the “placebo-effect” due to logistical challenges in conducting true blinded studies. Our understanding of the complex interplay between autonomic imbalance and HF is still evolving, and our ability to properly identify the patient population to derive maximum benefit from device-based neuromodulation is still very limited. There is still an unmet need for additional therapeutic strategies to improve outcomes in this vulnerable patient population.

Several other therapeutic strategies are under investigation which could revolutionize the field of neuromodulation in HF. Low-level tragus stimulation is a novel, noninvasive method of autonomic modulation via VNS, that has gained a lot of interest recently.^20^ Small studies have shown beneficial effects in terms of improvement in microcirculation and echocardiographic parameters in patients with HF.^21^^,^^114^ Further large trials are currently ongoing to evaluate the role of low-level tragus stimulation in patients with HF (Table 2).

Systemic inflammation plays a central role in pathophysiology of HF and is associated with higher mortality in patients with HF. Autonomic modulation has been proposed as a therapeutic target in reducing systemic inflammation, and autonomic modulation has been proposed as a potential therapeutic strategy aimed at reduction of systemic inflammation.^115^ Using these devices along with drug therapies can together play a significant role in improving patient outcomes and decrease the overall burden of disease.

## Conclusions

Device-based invasive and noninvasive neuromodulation techniques continue to hold promise in patients with HF phenotypes. The field of neuromodulation is quite complex and challenging and further research is warranted to better understand the impact of each of these therapies on the brain-heart-periphery axis and the effects on adrenergic tone, systemic inflammation, and cardiac intrinsic autonomic nervous system activity. The invasive nature of several of these aforementioned techniques does pose a hindrance to further development and acceptance, and therefore strategies need to be redirected to improve safety and tolerability and perhaps focus our efforts on evolving noninvasive neuromodulation techniques.
